# The Role of Gut Microbiome Perturbation in Fatigue Induced by Repeated Stress from Chemoradiotherapy: A Proof of Concept Study

**DOI:** 10.1155/2020/6375876

**Published:** 2020-02-07

**Authors:** Velda J. González-Mercado, Josué Pérez-Santiago, Debra Lyon, Israel Dilán-Pantojas, Wendy Henderson, Susan McMillan, Maureen Groer, Brad Kane, Sara Marrero, Elsa Pedro, Leorey N. Saligan

**Affiliations:** ^1^College of Nursing, University of South Florida, Tampa, FL, USA; ^2^Puerto Rico Omics Center, Comprehensive Cancer Center, University of Puerto Rico, San Juan, PR, USA; ^3^College of Nursing, University of Florida, Gainesville, FL, USA; ^4^Intramural Program, National Institute of Nursing Research/National Institute of Health, Bethesda, MD, USA; ^5^College of Arts and Sciences, University of South Florida, Tampa, FL, USA; ^6^School of Pharmacy, Medical Science Campus, University of Puerto Rico, San Juan, PR, USA

## Abstract

**Objectives:**

The objectives of this proof of concept study were to (a) examine the temporal changes in fatigue and diversity of the gut microbiome over the course of chemoradiotherapy (CRT) in adults with rectal cancers; (b) investigate whether there are differences in diversity of the gut microbiome between fatigued and nonfatigued participants at the middle and at the end of CRT; and (c) investigate whether there are differences in the relative abundance of fecal microbiota at the phylum and genus levels between fatigued and nonfatigued participants at the middle and at the end of CRT.

**Methods:**

Stool samples and symptom ratings were collected prior to the inception of CRT, at the middle (after 12–16 treatments) and at the end (after 24–28 treatments) of the CRT. Descriptive statistics and Mann–Whitney *U* test were computed for fatigue. Gut microbiome data were analyzed using the QIIME2 software.

**Results:**

Participants (*N* = 29) ranged in age from 37 to 80 years. The median fatigue score significantly changed at the end of CRT (median = 23.0) compared with the median score before the initiation of CRT for the total sample (median = 17.0; *p* ≤ 0.05). At the middle of CRT, the alpha diversity (abundance of Operational Taxonomic Units) was lower for fatigued participants (149.30 ± 53.1) than for nonfatigued participants (189.15 ± 44.18, *t*(23) = 2.08, *p* ≤ 0.05). At the middle of CRT, the alpha diversity (abundance of Operational Taxonomic Units) was lower for fatigued participants (149.30 ± 53.1) than for nonfatigued participants (189.15 ± 44.18, *Proteobacteria*, *Firmicutes*, and *Bacteroidetes* were the dominant phyla for fatigued participants, and *Escherichia*, *Bacteroides*, *Faecalibacterium*, and *Oscillospira* were the most abundant genera for fatigued participants.

**Conclusion:**

CRT-associated perturbation of the gut microbiome composition may contribute to fatigue.

## 1. Introduction

Locally advanced rectal cancer (RC) is treated with concomitant chemotherapy and radiation therapy (CRT) with curative intent [1]. Standard RT is delivered five days a week at doses of 1.8 Gy daily for a total of 50.4 Gy joined with either continuous of 5-fluorouracil (5-FU) over 5 days or oral capecitabine 5 days per week for a total of 5 weeks. Standard radiation therapy oncology group (RTOG) fields are used, which encompass the rectum, regional nodes, and pelvic small bowel. Although CRT has improved local control of RC and survival, it often produces disruptive side effects such as fatigue with negative impacts on health-related quality of life [2]. The etiology and associated mechanism of the cancer-related fatigue during CRT treatment remain elusive [3]. There is some evidence, however, that suggests that cancer treatment-induced gut microbial perturbation/dysbiosis (an imbalance in the intestinal microbiota or microorganism that live in the gut) contributes to inflammation-enabling translocation of bacteria and microbially-mediated metabolites into systemic circulation and inducing aberrant activation of the immune system such as cytokine-induced (i.e., interleukin-6) inflammatory reaction, which can affect brain function and induce behavioral symptoms such as fatigue [4–8]. However, not enough microbiomic studies exist to identify associations between changes of the gut microbiota and fatigue severity during CRT for RC. Fatigue phenotyping focused on gut microbiome perturbation can be a first step towards a better understanding of the biology of CRT-associated fatigue and the development of personalized medicine/interventions to target phenotype-based characteristics.

There is recent clinical evidence [9, 10] that RT or chemotherapy can induce perturbation of the gut microbiome diversity and composition as seen in mice studies [9, 11]. A study (*n* = 9) in gynecological cancer patients reported a significant decrease in the Shannon diversity index (bacterial species diversity) through the pelvic RT treatment [12]. In addition, they reported that during RT, the phylum *Fusobacterium* significantly increased, while the phylum *Firmicutes* decreased compared with before RT. One animal study (using real-time PCR to quantify bacteria) showed that 5-FU chemotherapy is associated with a decrease in *Clostridium* spp., *Lactobacillus* spp., and *Streptococcus* spp. and an increase in *Escherichia* spp., as well as a decrease of goblet cell numbers or mucus secretion in the jejunum of rats [13]. The aforementioned evidence suggests that patients undergoing chemotherapy or RT may exhibit marked changes in their intestinal microbiota [4, 5, 9]. However, these studies focused on chemotherapy or RT, not both, had heterogeneous groups and small sample sizes suggesting that more research is needed regarding the disruption of the intestinal microbiata during CRT.

Perturbation of the gut microbiota has been linked to worsening of fatigue during pelvic RT [8, 14]. One small cohort study found that fatigue symptoms were positively correlated with the systemic markers of inflammation (haptoglobin), and the microbial diversity, richness, and the *Firmicutes*/*Bacteroidetes* ratio was significantly altered prior to pelvic RT in patients who later developed diarrhea with significant worsening in fatigue [14]. There is an emerging interest in how CRT can play a part in decreased microbial diversity and increased bloom of pathobionts to influence systemic inflammatory responses with consequential behavioral effects [4, 5, 8, 14]. This pilot study is aligned with that interest. Therefore, the objectives of this proof of concept study were to: (a) examine the temporal changes in fatigue scores and in diversity of the gut microbiome over the course of CRT of adults with RC; (b) investigate whether there are differences in diversity of the gut microbiome between fatigued and nonfatigued participants at the middle and at the end of CRT; and (c) investigate whether there are differences in the relative abundance of fecal microbiota, at the phylum and genus levels, between fatigued and nonfatigued participants at the middle and at the end of CRT. Further research may point to a possible microbial signature that can identify RC patients at risk to develop acute CRT-related fatigue. Further, a better stratification of at-risk patients may optimize clinical care as well as improve quality of life and health outcomes of patients and families.

## 2. Materials and Methods

### 2.1. Study Population

Newly diagnosed RC patients scheduled to receive CRT were recruited for this study. Exclusion criteria included progressive or unstable disease of any body system causing clinically significant fatigue; documented history of major depression within the past 5 years; and uncorrected hypothyroidism or anemia. Patients taking antibiotics, prebiotics, probiotics, steroids, and/or immune-suppressant agents within one month prior to any event of sample collection were also excluded. Data collection was conducted from September 2017 to April 2018. The recruitment and data collection of study participants took place at three ambulatory cancer treatment facilities: two were in the southeastern United States, and one was in San Juan, Puerto Rico. The Human Subjects Committees of both the Southeastern Academic Medical Center and University of Puerto Rico Medical Science Campus approved the study prior to data collection. All procedures performed in studies involving human participants were in accordance both with the ethical standards of the institutional and/or national research committee and with the 1964 Helsinki Declaration and its later amendments or comparable ethical standards. Written informed consent was obtained from all individual participants included in the study.

### 2.2. Demographics, Clinical Information, and Assessment of Fatigue

After obtaining informed consent, participants completed 2 paper forms: demographic information and the 7-item Patient-Reported Outcome Measures Information System-fatigue (PROMIS-F). The forms were administered before, at the middle (after 12 to 16 CRT treatments), and at the end (after 24–28 treatments) of the CRT. The demographic form collected the respondent's age, marital status, occupational status, and education level. The principal investigator obtained the following clinical information from the medical chart: stage of cancer, before treatment hemoglobin (Hgb) levels, prescribed and self-administered medications, number of RT fractions, and total RT dose.

The 7-item, adult PROMIS-F form assesses the impact and experience of fatigue in the past week [15]. For example, one of the items reads “in the past seven days, how often did you feel tired?” Each item was anchored by a 5-point Likert-type scale response (1 = never to 5 = always). Scores ranged between 7 and 35 where high scores mean higher fatigue. A total score was calculated by summing scores across items. The 7-item PROMIS-F, Spanish and English versions have been widely used to evaluate cancer-related fatigue and have well-documented validity and reliability [15–17]. The PROMIS-F internal consistency in the present study was 0.90. Further, we compared the mean raw scores using the recommended raw score meaningfully important differences (MIDs) ranges for research in cancer patients which suggest that raw-score MIDs estimates for the 7-item PROMIS-F are in the range of 2-3 [17]. To optimize the fatigue phenotypic characterization of the study participants, we grouped participants for analysis based on a 3-point change in PROMIS-F score from before to middle and from before to end of CRT; fatigued participants were those with ≥3-point increase in PROMIS-F score, and nonfatigued participants were those with <3-point change in fatigue score.

### 2.3. Fecal Samples Collection

On the same day of the fatigue assessment (before, at the middle, and at the end) of CRT, each participant collected approximately 5 g of stool using a sterile plastic container for each of the three study time points. Participants stored the stool samples at their home refrigerator and delivered them in person within 24 hr to the research team. Aliquots of 250 mg of stool samples received were immediately stored at −80°C for approximately 30 days before batch DNA extraction.

### 2.4. 16s RNA Gene Sequencing Microbiome Assay

Extraction of DNA from stool bacteria (from stool samples before (*n* = 29), at the middle (*n* = 26), and at the end (*n* = 22) of CRT) for 16S RNA amplicon sequencing was conducted following the Power Soil DNA Isolation kit (MoBio, Carlsbad, CA) procedure and used as the template for PCR amplification. The differences in number of stool samples at each time point was due to either missing stool specimens or antibiotics or probiotics being used at time of collection. DNA concentrations were assessed by Qubit 3.0 Fluorometer (Life Technologies; Thermo Scientific, Wilmington, DE). DNA integrity was evaluated by loading 5 µl DNA on a 1% agarose gel stained with ethidium bromide. Microbial sequencing was performed on the MiSeq Illumina platform following the Illumina protocol [18]. The V3/V4 region of the 16S bacterial rDNA was amplified using the standard Illumina primers. Reverse primers included a 12 base pairs (bp) nucleotide barcode to facilitate multiplex sequencing of all 85 samples on a single 300 bp paired-end MiSeq run (V3 kit). All reads that passed the initial quality control were kept for further analyses, and the adapter and barcode sequences were removed. Both read-ends were merged using a paired-end read merger (PEAR) with a minimum overlap of 20 bp and a significance of 0.001 [19]. We obtained a total of 14,430,624 reads with an average of 169,773 reads per sample.

The diversity measurements and microbial taxonomic classification were performed using Quantitative Insights into Microbial Ecology (Qiime) version 2 software. Following the Qiime pipeline, high-quality Illumina reads were trimmed and denoised using DADA2 [20], and then the remaining sequences were aligned using Mafft [21]. A phylogenetic tree was built to calculate metrics of Alpha diversity: Shannon diversity index (accounts for both abundance and evenness of the species present), number of observed Operational Taxonomic Units (OTUs), Pielou's evenness, and Faith phylogenetic diversity index (the estimators of microbial community evenness). In addition, taxonomic classification at the phylum and genus levels was done with the qiime2 feature classifier plugin using the Greengenes 16S database and the amplification primers used to select the V3-V4 region. All diversity metrics and the relative abundance were utilized to investigate effects of CRT on the gut microbiome and its association with fatigue.

### 2.5. Statistical Analysis

Descriptive statistics including the frequency, percentages, means, and standard deviations (SDs) were performed on demographics and disease characteristics of the sample. In addition, descriptive statistics including the means, SDs, and medians were computed for the PROMIS-F for the sample as a whole and by fatigue group categories. Because histograms showed that the PROMIS-F data were not normally distributed, a nonparametric (Mann–Whitney *U* test) statistical test was used to evaluate differences between before to middle and between before to end of CRT. We used a Chi-squared test or Spearman's correlation to investigate the possible association between fatigue and (a) infusion chemotherapy vs. oral chemotherapy, (b) stage, (c) gender, (d) age, (3) BMI (body mass index), and (f) before treatment Hgb levels. The data were analyzed using Statistics Package for Social Sciences SPSS, version 24.0 for windows, and/or the R statistical software.

Normality of the gut microbiome variables (diversity and relative abundance) was assessed using a Shapiro test with a significance threshold of *p* ≤ 0.05. We used mixed-effects regression analyses to investigate differences in the gut microbiome diversity metrics over the course of the CRT while adjusting for repeated measurements. We used a double-tailed *t*-test or a Mann–Whitney *U* test with a significance level of ≤0.05 to assess statistical differences between groups based on fatigue status at the middle and end of CRT. To test whether microbiome variables (diversity and taxonomy) before CRT could potentially predict fatigue status after treatment, we used random forest classification. We assessed performance of classification using balance accuracy, defined as the average between sensitivity (proportion of fatigue individuals correctly identified) and specificity (proportion of nonfatigue individuals correctly identified). Variable importance for classification was assessed using the mean decrease in the Gini index.

## 3. Results

### 3.1. Patient Characteristics

We approached 36 men and women for possible participation in the study; 33 agreed to participate, but only 29 were eligible and were consented. The mean age of study participants was 61 years (SD 11.4); the large majority was married or partnered (96%), well educated (85%), and retired (69%). Most of the participants were male (nearly 61%) and non-Hispanic Whites (48%) followed by Hispanics (30%).  Table 1 describes the clinical characteristics of the participants. Twenty participants had a clinical stage of T3 (69%), and 9 patients had stage 2 RC. Patients were treated with the 3D technique with a standard protocol of 45 Gy in 25 fractions to the pelvis, including the posterior pelvic nodes, rectum, and mesorectum up to the L5-S1 disk space, and then a 6 to 8 Gy boost (51 to 53 Gy total) in 3 or 4 fractions to the original tumor volume with a 1.5 cm margin. A total of 17 patients (53%) received concurrent continuous infusion of 5-FU 225 mg/m^2^ over 24 hours, and 12 patients received oral capecitabine 825 mg/m^2^ twice a day, 5 days per week for a total of 5 weeks. Before treatment, Hgb levels were within reference ranges (Table 1).

### 3.2. Severity of CRT-Related Fatigue

There were significant differences in severity of fatigue at the end of CRT (higher scores indicate more severity) compared with the median scores before the initiation of CRT (*p* ≤ 0.05) (Table 2). Half of the participants were fatigued (*n* = 13; median fatigue = 19) at the middle of the CRT, while half (*n* = 13; median fatigue = 20) were in the nonfatigue group. Further, at the end of the CRT, most participants were in the fatigue group (*n* = 15; median fatigue = 24), and 7 were in the nonfatigue group (median fatigue = 18). There were no statistical association between fatigue status and infusion chemotherapy vs. oral chemotherapy neither at middle nor at end of treatment (Chi-squared, *p* > 0.05). Similarly, there were no significant association between fatigue status and gender (*p* > 0.05) at any time point. Furthermore, while there were no associations between fatigue scores at any time point with either treatment, age, BMI, number of treatment or before treatment Hgb levels (*p* > 0.05), higher fatigue scores at before treatment (rho = 0.50, *p*=0.001) and at the middle of treatment (rho = 0.45, *p*=0.001) were associated with higher stage of disease, respectively.

### 3.3. Temporal Changes in Diversity of the Gut Microbiome over the Course of CRT for the Total Sample Reads with an Average of Reads per Sample

**Figure 1:**
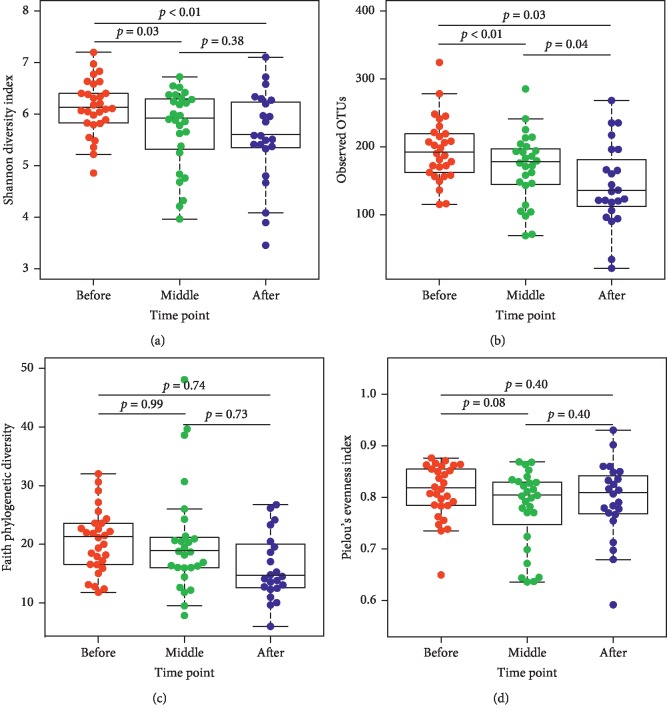
Alpha diversity indexes across time points of treatment (before (*n* = 29), middle (*n* = 26), and end (*n* = 22)) for all participants: (a) Shannon, (b) observed OTUS, (c) Faith, and (d) Pielou.

Table 3 shows the median values for each computed alpha diversity indexes, namely, (a) Shannon's diversity; (b) number of observed OTUs; and (c) the estimators of community evenness (Faith and Pielou). Further comparison analysis for alpha diversity using the mixed-effects regression analyses showed a significantly lower Shannon's diversity at middle (*p* ≤ 0.03) and at the end of CRT (*p* < 0.01) compared with before treatment for the whole sample (Figure 1(a)). Similarly, a significantly lower abundance of OTUs at the middle (*p* ≤ 0.01) and at the end of treatment were found (*p* ≤ 0.03) compared with the before treatment OTUs (Figure 1(b)). There is also a trend towards lower Pielou's evenness index at the middle of CRT compared with before the initiation of treatment (Figure 1(d)). There were no significant differences in any of the diversity indexes between those on infusion of 5-FU vs. those on oral capecitabine at the middle nor at the end of CRT, which suggests that both groups are equivalent with respect to chemotherapy ([Supplementary-material supplementary-material-1]. Table).

### 3.4. Differences in Alpha Diversity Indexes between Fatigued and Nonfatigued Participants across Treatment

**Figure 2:**
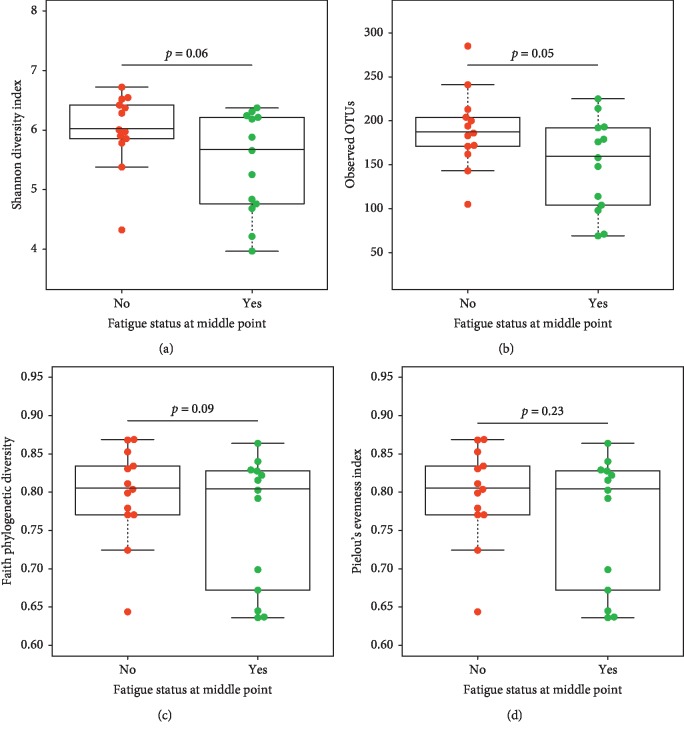
Comparison of alpha diversity indexes at the middle of CRT for participants in each fatigue group (No = nonfatigued (*n* = 13); Yes = fatigued (*n* = 13)): (a) Shannon, (b) observed OTUs, (c) Faith, and (d) Pielou.

We compared the alpha diversity index profiles between fatigued and nonfatigued participants at the middle and at the end of CRT. Follow-up tests using Welch's *t*-test showed significant differences between fatigued and nonfatigued patients at the middle of treatment (Figure 2(b)). More specifically, the mean abundance of OTUs was lower for fatigued patients (149.30 ± 53.1) than for nonfatigued patients (189.15 ± 44.18, *t*(23) = 2.08, *p* ≤ 0.05). A similar trend was observed for the Shannon and Faith diversity indexes at the middle of CRT (Figures 2(a) and 2(c)). However, at the end of CRT, there were no significant differences for any alpha diversity indexes between fatigued and nonfatigued patients, which is likely due to the small sample size of this exploratory study ([Supplementary-material supplementary-material-1]. Table).

### 3.5. Differences in Relative Abundance of Most Common Phylum between Fatigued and Nonfatigued Participants across Treatment

With taxonomic analysis, *Firmicutes* and *Bacteroidetes* were the two largest phyla for both the fatigued and nonfatigued participants in the middle and at the end of CRT, respectively (Table 4). In the middle of treatment, there is a significantly lower relative abundance of *Bacteroidetes* in fatigued participants compared with nonfatigued participants (*U* = 124, *p* ≤ 0.04) (Table 4). In the middle of the CRT, a similar trend was observed in fatigued participants showing an increase in Firmicutes/Bacteroidetes ratio compared with nonfatigued participants. At the end of treatment, there is a significantly higher relative abundance of *Bacteroidetes* in fatigued participants compared with nonfatigued participants (*U* = 16, *p* ≤ 0.01). There is also a trend towards a lower abundance of *Firmicutes/Bacteroidetes* ratio and *Firmicutes* among fatigued participants compared with nonfatigued participants.

### 3.6. Differences in Relative Abundance of Selected Genera between Fatigued and Nonfatigued Participants across Treatment

#### 3.6.1. Fecal Microbial Ecology of Fatigued Participants

**Figure 3:**
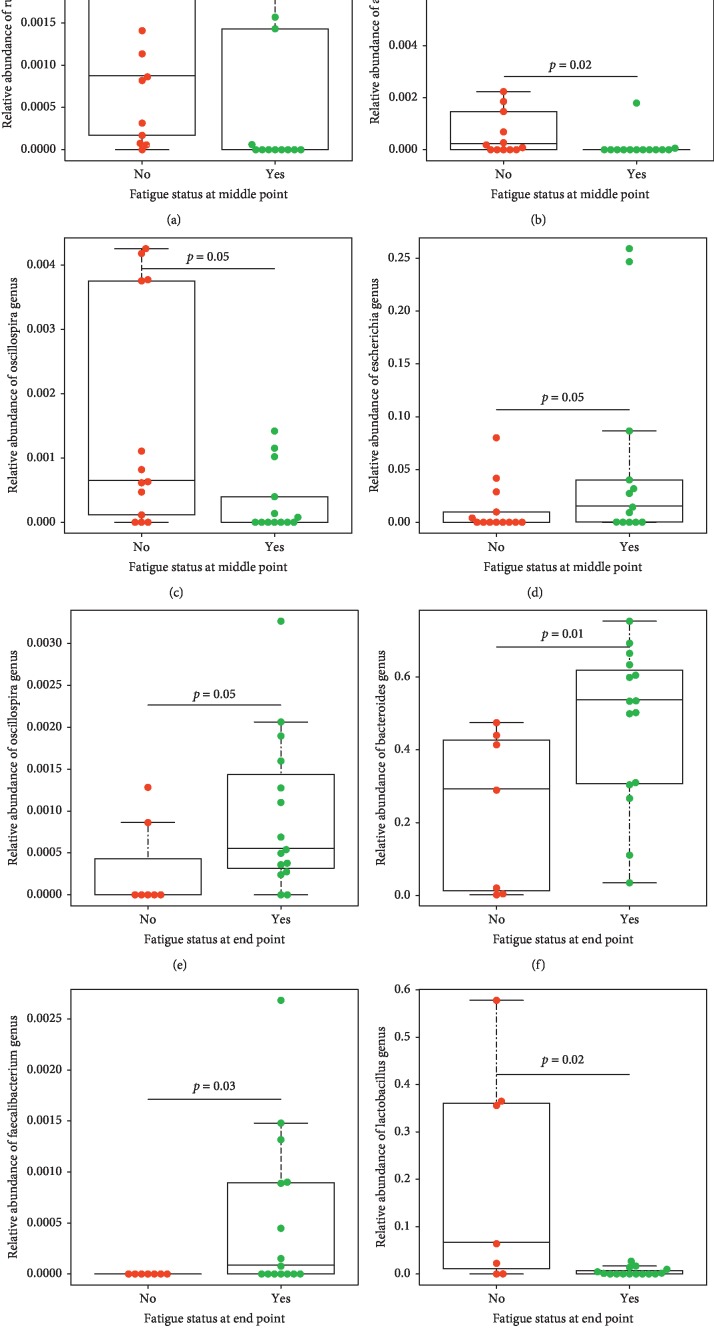
Boxplots showing the relative abundance of selected genera between fatigued and nonfatigued groups (No/= nonfatigued; Yes = fatigued) based on Mann–Whitney *U* test in fecal samples at the middle of treatment ((a) *Ruminococcus*, (b) *Akkermansia*, (c) *Oscillospira*, and (d) *Escherichia* genus) and at the end of treatment ((e) *Oscillospira*, (f) *Bacteroides*, (g) *Faecalibacterium*, and (h) *Lactobacillaceae*) relative to baseline, pre-CRT. Middle of treatment (fatigued *n* = 13; nonfatigued *n* = 13); end of treatment (fatigued *n* = 15; nonfatigued *n* = 7).

The fecal microbial composition between fatigued and nonfatigued participants across treatment was also significantly different at the genus level (Figure 3). More specifically, we observed that at the middle of treatment, the fatigued participants' microbiota was significantly enriched from the *Escherichia* genus belonging to the phylum of *Proteobacteria* compared with nonfatigued participants (little to no representation of this genus) (*p* ≤ 0.05) (Figure 3(d)). At the end of treatment, the fatigued participants' microbiota was significantly enriched from genera *Bacteroides* (Figure 3(f)), which belong to the *Bacteroidetes* phylum, and members of the *Firmicutes* phylum, including the *Faecalibacterium* and the *Oscillospira* genera compared with nonfatigued participants (*p* ≤ 0.05) (Figures 3(e) and 3(g)).

#### 3.6.2. Fecal Microbial Ecology of Nonfatigued Participants

At the middle of treatment, we observed that the nonfatigued participants' microbiota was significantly enriched by a member belonging to the *Verrucomicrobia* phylum, the *Akkermansia* genus (Figure 3(b)) and by members of the *Firmicutes* phylum, including members of the *Ruminococcaceae* family, such as the *Ruminococcus* and the *Oscillospira* genera (Figures 3(a) and 3(c)) compared with the microbiota of fatigued participants (*p* ≤ 0.05). At the end of treatment, we observed that the nonfatigued participants' microbiota was significantly enriched from members of the *Firmicutes* phylum, including members of the *Lactobacillaceae* family such as *Lactobacillus* genus (*p* ≤ 0.05) (Figure 3(h)).

#### 3.6.3. Testing Whether Microbiome Variables (Diversity and Taxonomy) before CRT Could Potentially Predict Fatigue Status during CRT

**Figure 4:**
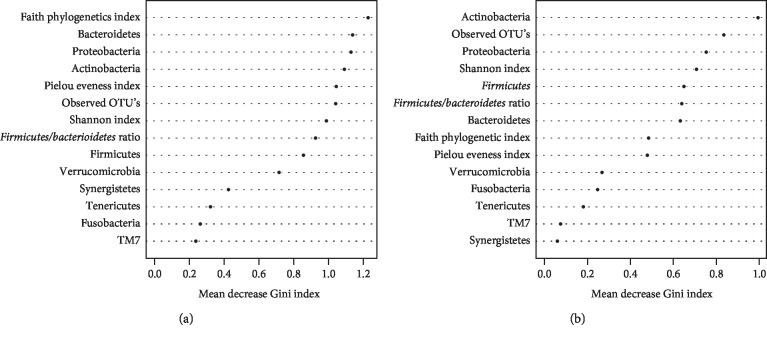
Variable importance for classification of fatigue status by random forest at the (a) middle of CRT and (b) end of CRT.

We performed random forest classification using the baseline microbiome variables as predictors and fatigue status at the middle and at the end of treatment separately as outcomes (Figure 4). Microbiome variables included Shannon diversity index, OTUs, Pielou's evenness index, Faith phylogenetic diversity index, the 9 taxonomic assignments at the phylum level (*Proteobacteria*, *Actinobacteria*, *Bacteroidetes*, *Firmicutes, Verrucomicrobia*, *Fusobacteria*, *Tenericutes*, *TM7*, and *Synergistetes*), and the *Firmicutes/Bacteroidetes* ratio. At the middle point, we had 63% balanced accuracy in predicting participants with and without fatigue with a sensitivity of 70% and specificity of 57%. At the end of treatment, we had 49% balanced accuracy in predicting participants with and without fatigue with a sensitivity of 73% and specificity of 25%.

## 4. Discussion

Our findings and discussion must be treated as preliminary and should be interpreted with caution because of the several limitations discussed below. Nonetheless, fatigue often has been reported as one of the most distressing symptoms that cancer patients face during treatment. In fact, evidence from cross-sectional studies suggests a prevalence of severe fatigue in approximately 67% of patients during neoadjuvant CRT for nonmetastatic RC [22], and 85% of patients complained of persistent fatigue post-CRT [23]; however, limited longitudinal studies are available. The results of this exploratory study showed that fatigue worsened significantly from before to the end of CRT (*p*=0.006 − 0.03). Similar findings have been reported among head and neck cancer patients undergoing CRT [24]. Interestingly, we did not find that fatigue was associated with type of chemotherapy, gender, age, BMI, nor with before treatment Hgb levels. These findings are consistent with other reports in which neither gender and age nor total dose of RT predicted fatigue severity during RT for patients with different cancer diagnoses [25]. Similarly, Gurren et al. [26] showed that there was no correlation between fatigue scores and Hgb levels during neoadjuvant RT for RC. The contrary is found in a sample of advanced cancer patients admitted for symptom control in which general fatigue was correlated with Hgb levels [27]. Nonetheless, given the high association of tumor stage with fatigue, clinicians need to be aware of the possibility of worsening of fatigue among those who present with higher tumor stage. These findings also suggest the need for clinicians to continuously assess, plan, and manage fatigue and other potential side effects of CRT in order to improve treatment outcomes in this understudied population [2]. A major gap in preventing and managing CRT-induced fatigue is that its biological mechanisms have not been identified. Nonetheless, renewed interest in the gut-brain-axis and the notable advances in high dimensional sequencing techniques/data have suggested a role of CRT-induced gut microbial perturbation/dysbiosis in the pathobiology of CRT-related fatigue [4, 5, 8, 9, 14]; however, comprehensive microbiomics studies in this clinical population are scarce.

Our findings of this exploratory study suggest that various factors such as CRT resulted in perturbation of the gut microbiome composition. More specifically, gut microbial perturbation in this study was characterized by within-sample reduction in diversity in the middle and at the end of CRT compared with before treatment for both the Shannon diversity index and the observed species/OTUS. Similar findings have been reported in patients undergoing chemotherapy or pelvic RT alone. One study using 454 high-throughput pyrosequencing data found that both alpha diversity indexes (Faith's phylogenic diversity and observed species) in 15 fecal samples collected after chemotherapy for non-Hodgkin's lymphoma were significantly lower compared with samples collected before treatment [28]. Similarly, a small study (*n* = 11) among mixed pelvic cancer patients, using 454 high-throughput pyrosequencing, found a significant decrease in the Shannon diversity index after pelvic RT compared with before RT [14]. Interestingly, one cross-sectional study compared the fecal microbiota of 33 healthy individuals, 17 colorectal cancer (CRC) patients before treatment, 14 CRC patients treated with chemotherapy, and 5 CRC patients after surgery, but found no significant differences in alpha diversity indexes among groups [29]. Nonetheless, our study findings suggest that the global balance of bacterial populations in the gut changed in response to genotoxic stress such as CRT.

Our investigation of the diversity of bacteria over time revealed the novel finding that at the middle of CRT, the observed OTUS diversity was lower among fatigued participants than nonfatigued participants. As previously highlighted, one pilot study suggested that gut microbiota perturbation/dysbiosis might predict diarrhea and worsening of fatigue in cancer patients receiving pelvic RT [14]. Other authors have attempted to show a relationship between microbial diversity and behavioral symptoms in other fatiguing conditions (e.g., myalgic encephalomyelitis/chronic fatigue syndrome (ME/CFS)) [30]. For example, one study reported a reduced diversity and altered composition of the gut microbiome in individuals with ME/CFS compared with healthy individuals [30]. Further longitudinal studies should identify if decreased gut microbial diversity or time during treatment are the most significant drivers of CRT-associated fatigue.

Our pilot taxa analysis results found that at the middle of CRT, the majority of bacteria present in the gut of fatigued participants was represented by *Proteobacteria* from the *Escherichia* genera, which may include a potential pathogen such as *E. coli*. *E. coli* and *Salmonella* commonly proliferate in the blood cultures of hospitalized cancer patients with fever [31]. Some strains of *E. coli* particularly in immunocompromised cancer patients have been linked to potentially severe and life-threatening infections such as UTI [32], both acute and chronic diarrhea (e.g., Enteropathogenic and enterohaemorrhagic *E. coli* diarrhea [33], or bloodstream infections) [34]. For example, one study (using real-time PCR to quantify bacteria) observed that the microflora composition of the majority of patients experiencing chemotherapy-induced diarrhea showed increase in *E. coli* and a decrease in *Lactobacillus* spp. and *Bacteroides* spp. compared with healthy voluntaries [33]. Also, some strains of *E. coli* are positively correlated with CRC tumor size [35]. In addition, some evidence suggests associations between *E.coli* and a compromise intestinal barrier integrity, inflammation, and potential hepatotoxic events in nonalcoholic fatty liver disease [36]. This is remarkable because perhaps our data support the hypothesis of a proinflammatory environment in the fatigued group as evidenced by a significantly higher relative abundance of both the *Bacteroidetes* phylum and the genera *Bacteroides* compared with the nonfatigued participants at the end of CRT. At the phylum level, increased *Bacteroidetes* have been reported in intestinal inflammatory conditions such as irritable bowel syndrome [37] and nonalcoholic fatty liver disease [36]. Interestingly, the *Bacteroides* genera may include a potential pathogen such as the *B. fragilis*. In fact, a recent review of the literature compiled information suggesting that enterotoxigenic strains of *B. fragilis* have not only been associated with CRC but also with bacteremia, colitis, diarrhea, sepsis, systemic infection, systemic inflammation, and neurological diseases such as Alzheimer's disease [38]. This latter review also highlighted that one of the mechanisms by which *B. fragilis* can be associated with systemic inflammation and neurodegeneration may be related to the ability of *B. fragilis* to secrete neurotoxins such as proinflammatory lipopolysaccharide BF-LPS. On the contrary, some strains of *Faecalibacterium* (which was also significantly abundant among fatigued participants) such as *F. prausnitzii* [39] and some strains of *Oscillospira* [40] have been linked to anti-inflammatory properties.

We also demonstrated that at the middle of treatment, the majority of bacteria present in the gut of nonfatigued participants was represented by two distinct phyla: the *Verrucomicrobia* phylum (*Akkermansia* genus, a beneficial microbe) and the *Firmicutes* phylum (*Ruminococcus* genera and *Oscillospira* genera). This is important given that *Akkermansia* has been reported to lead to anti-inflammatory action in the intestinal tract and the enhancement of the intestinal barrier function [41]. More specifically, it has been suggested that *A. muciniphila* adheres to enterocytes and strengthens the integrity of the epithelial cell layer, resulting in a favorable intestinal immunity [42]. Further, butyrate-producing genera such as *Oscillospira* were detected among both, nonfatigued participants at the middle of treatment and fatigued participants at the end of treatment [40]. An interesting fact is that animal studies have shown that butyrate supplementation improves gut barrier function, by attenuating inflammation and reducing endotoxin levels [43]. It is noteworthy that our study also identified that nonfatigued participants at the end of treatment showed a *Lactobacillus* predominance in stool samples, perhaps representing a period of clinical stability. The order *Lactobacillales* includes species (e.g., *Lactobacillus*, *Lactococcus*, and *Enterococcus* species) that have been linked to the production of an acidic environment that inhibits the growth of several species from harmful bacteria [44]. Also, some strains in the genus *Lactobacillus* (e.g., *L. acidophilus, L. casei*) have been found to have probiotic properties [44], to ameliorate gut inflammation [45], and to improve gut barrier function [46] in animal models. On the contrary, the abundance of *Oscillospira* and *Ruminococcus* has been linked to aging [47]. Notably, comprehensive molecular analyses studies are needed to confirm and determine the role of a certain group of bacteria in the etiology of CRT-associated fatigue and other co-occurring symptoms (e.g., sleep disturbance, depression, anxiety, pain, and cognitive impairment) as well as in CRT-induced gastrointestinal symptoms (e.g., mucositis, nausea, and vomiting) [13, 33].

In contrast to our expectations, our preliminary study found that gut microbial variables prior CRT have lower accuracy in predicting fatigue during the acute phase of treatment. This lower accuracy could be explained in part by our moderate sample size. It may also be that other variables that may have had a stronger association with fatigue (e.g., depression, albumin levels, inflammatory markers (e.g., haptoglobin, interleukin-1 receptor antagonist, soluble tumor necrosis factor receptor2 levels), and uncontrolled diarrhea, [14, 22, 27, 48]) were not included in the analysis. Nonetheless, our study sets the stage for future validation and optimization of the classifier/model in a larger sample, perhaps including a subgroup of healthy participants together with clinical data in a well-designed longitudinal study. The ability to differentiate between patients with and without fatigue symptoms or having a particular microbiome that may be risky for developing symptoms has clinical relevance for the development of personalized medicine/interventions.

### 4.1. Limitations

Limitations of this exploratory study include the use of fecal samples and not intestine contents. However, fecal samples are commonly used to study microbial communities [49], perhaps because these samples are easy to obtain from individuals compared with intestinal tissue samples. Another limitation of this study is the relatively moderate sample size; a larger sample size would have permitted examining other variables of interest. For example, there may be other side effects/variables caused by the cytotoxic effects of CRT (e.g., anemia, nutritional status [50], gastrointestinal disturbances (mucositis, diarrhea and constipation; [39]), or external factors (e.g., diet; [51]) that account for variance in both the composition and diversity of gastrointestinal bacterial populations that may influence the fatigue experience that were not included in the analysis; however, collection of data on those variables is currently ongoing. Longer longitudinal studies examining persistent fatigue and dysbiosis are needed to validate previous studies showing that after the end of chemotherapeutic administration; the bacterial abundance recovered within a few days, sometimes even with numbers elevating above initial levels [39]. These findings could provide information regarding therapeutic efforts to ameliorate both fatigue and gut microbiota perturbation during the acute phase of treatment. In the context of RC, another limitation of this study was not having data on contributing factors, such as tumor characteristics (e.g., location and size [49]) or surgical recovery (e.g., ischemia/reperfusion [52]) which can influence the gut microbiome and the tumor microenvironment, in addition to other concomitant treatments (e.g., CRT).

## 5. Conclusions

In our pilot study, we were able to extend the knowledge about gut microbiota perturbation overtime during the course of CRT and its relationship with fatigue in order to provide a foundation for future mechanistic studies. Our preliminary results showed that *Proteobacteria*, *Firmicutes*, and *Bacteroidetes* were the dominant phyla for fatigued participants, and *Escherichia*, *Bacteroides*, *Faecalibacterium*, and *Oscillospira* were the most abundant genera for fatigued participants. One of the possible routes for correcting dysbiosis is by the administration of probiotics [44], which might be part of our future research.

## Figures and Tables

**Table 1:** Clinical characteristics of sample.

Variable	Mean	SD	Range	*N*	Reference range
Age	61.00	11.40	37–80	29	
Body mass index	27.19	4.16	15.5–38.0	26	
Hemoglobin	13.96	1.17	12.20–17.70	22	11–18 g/dL

SD = standard deviation.

**Table 2:** Summary of Mann–Whitney *U* test for the PROMIS-F for the total sample.

Mann–Whitney			
Pairs	Median	*U*	*p* value
Before vs.	17.0	307.5	0.17
Middle	20.0		
Before vs.	17.0	218.5	0.03^*∗*^
End	23.0		
Middle vs.	20.0	253.5	0.27
End	23.0		

^*∗*^ *p* ≤ 0.05.

**Table 3:** Medians for alpha diversity indexes before (*n* = 29), at the middle (*n* = 26), and at the end (*n* = 22) of CRT.

Index	Before	Middle	End
	Median	Median	Median
Observed			
OTUS	190	176	134
Shannon	6.11	5.90	5.58
Faith	21.14	18.70	14.52
Pielou	0.82	0.80	0.81

The differences in number of samples at each time point were due to either missing stool specimen or antibiotics or probiotic use at time of collection.

**Table 4:** Summary of Mann–Whitney *U* test for taxonomic analysis of relative abundance of four main bacterial phyla.

Phylum	Fatigued	Nonfatigued	Mann–Whitney *U*	
	Median	Median	*U*	*p* value
*Firmicutes/Bacteroidetes*				
Middle	0.74	0.32	50	0.08
End	0.22	1.33	79	0.07
Actinobacteria				
Middle	0.002	0.002	78	0.76
End	0.0008	0.0009	61	0.57
Bacteroidetes				
Middle	0.22	0.72	124	0.04^*∗*^
End	0.65	0.41	16	0.009^*∗∗*^
Firmicutes				
Middle	0.23	0.23	71	0.51
End	0.14	0.51	78	0.08
Proteobacteria				
Middle	0.13	0.05	61	0.24
End	0.09	0.23	54	0.94

^*∗*^ *p* ≤ 0.05 and ^*∗∗*^*p* ≤ 0.01 middle of treatment (fatigued *n* = 13; nonfatigued *n* = 13); end of treatment (fatigued *n* = 15; nonfatigued *n* = 7).

## Data Availability

The data used to support the findings of this study are available from the corresponding author upon request.

## Supplementary Materials

Supplementary Materials S1 Table: comparison of alpha diversity indexes at the middle and end of CRT for participants based on chemotherapy treatment; end of treatment (5-Fu n=13; oral capecitabine n=9). S2 Table: comparison of alpha diversity indexes at the end of CRT for participants in each fatigue group(nonfatigue and fatigued N=7 and N=15).

## References

[1] National Comprehensive Cancer Network Clinical practice guidelines in oncology. *Rectal Cancer*.

[2] O’Gorman C., Barry A., Denieffe S., Sasiadek W., Gooney M. (2016). Nursing implications: symptom presentation and quality of life in rectal cancer patients. *Journal of Clinical Nursing*.

[3] Saligan L. N., Olson K., Filler K. (2015). The biology of cancer-related fatigue: a review of the literature. *Supportive Care in Cancer*.

[4] Bajic J. E., Johnston I. N., Howarth G. S., Hutchinson M. R. (2018). From the bottom-up: chemotherapy and gut-brain Axis dysregulation. *Frontiers in Behavioral Neuroscience*.

[5] Jordan K. R., Loman B. R., Bailey M. T., Pyter L. M. (2018). Gut microbiota-immune-brain interactions in chemotherapy-associated behavioral comorbidities. *Cancer*.

[6] Ferreira M. R., Muls A., Dearnaley D. P., Andreyev H. J. N. (2014). Microbiota and radiation-induced bowel toxicity: lessons from inflammatory bowel disease for the radiation oncologist. *The Lancet Oncology*.

[7] Kelly D. L., Lyon D. E., Yoon S. L., Horgas A. L. (2016). The microbiome and cancer. *Cancer Nursing*.

[8] Jakobsson S., Ahlberg K., Taft C., Ekman T. (2010). Exploring a link between fatigue and intestinal injury during pelvic radiotherapy. *The Oncologist*.

[9] Touchefeu Y., Montassier E., Nieman K (2014). Systematic review: the role of the gut microbiota in chemotherapy- or radiation-induced gastrointestinal mucositis—current evidence and potential clinical applications. *Alimentary Pharmacology & Therapeutics*.

[10] Manichanh C., Varela E., Martinez C. (2008). The gut microbiota predispose to the pathophysiology of acute postradiotherapy diarrhea. *The American Journal of Gastroenterology*.

[11] Kim Y. S., Kim J., Park S.-J. (2015). High-throughput 16S rRNA gene sequencing reveals alterations of mouse intestinal microbiota after radiotherapy. *Anaerobe*.

[12] Nam Y.-D., Kim H. J., Seo J.-G., Kang S. W., Bae J.-W. (2013). Impact of pelvic radiotherapy on gut microbiota of gynecological cancer patients revealed by massive pyrosequencing. *PLoS One*.

[13] Stringer A. M., Gibson R. J., Logan R. M. (2009). Gastrointestinal microflora and mucins may play a critical role in the development of 5-Fluorouracil-induced gastrointestinal mucositis. *Experimental Biology and Medicine*.

[14] Wang A., Ling Z., Yang Z. (2015). Gut microbial dysbiosis may predict diarrhea and fatigue in patients undergoing pelvic cancer radiotherapy: a pilot study. *PLoS One*.

[15] National Institutes of Heath (December 2018). PROMIS-fatigue. http://www.nihpromis.org/Science/PubsDomain/Fatigue_adult.aspx?AspxAutoDetectCookieSupport=1.

[16] Rothrock N. E., Hays R. D., Spritzer K., Yount S. E., Riley W., Cella D. (2010). Relative to the general US population, chronic diseases are associated with poorer health-related quality of life as measured by the Patient-Reported Outcomes Measurement Information System (PROMIS). *Journal of Clinical Epidemiology*.

[17] Yost K. J., Eton D. T., Garcia S. F., Cella D. (2011). Minimally important differences were estimated for six Patient-Reported Outcomes Measurement Information System-Cancer scales in advanced-stage cancer patients. *Journal of Clinical Epidemiology*.

[18] Illumina Inc. (2015). 16S rRNA Amplification Protocol version 4_13. Personal email communication.

[19] Zhang J., Kobert K., Flouri T., Stamatakis A. (2014). PEAR: a fast and accurate Illumina Paired-End reAd mergeR. *Bioinformatics*.

[20] Callahan B. J., McMurdie P. J., Rosen M. J., Han A. W., Johnson A. J. A., Holmes S. P. (2016). DADA2: high-resolution sample inference from Illumina amplicon data. *Nature Methods*.

[21] Katoh K., Standley D. M. (2013). MAFFT multiple sequence alignment software version 7: improvements in performance and usability. *Molecular Biology and Evolution*.

[22] Wang X. S., Janjan N. A., Guo H. (2001). Fatigue during preoperative chemoradiation for resectable rectal cancer. *Cancer*.

[23] Gosselin T. K., Beck S., Abbott D. H. (2016). The symptom experience in rectal cancer survivors. *Journal of Pain and Symptom Management*.

[24] Rosenthal D. I., Mendoza T. R., Fuller C. D. (2014). Patterns of symptom burden during radiotherapy or concurrent chemoradiotherapy for head and neck cancer: a prospective analysis using the University of Texas MD Anderson Cancer Center Symptom Inventory-Head and Neck Module. *Cancer*.

[25] Hickok J. T., Roscoe J. A., Morrow G. R., Mustian K., Okunieff P., Bole C. W. (2005). Frequency, severity, clinical course, and correlates of fatigue in 372 patients during 5 weeks of radiotherapy for cancer. *Cancer*.

[26] Guren M. G., Dueland S., Skovlund E., Fosså S. D., Poulsen J. P., Tveit K. M. (2003). Quality of life during radiotherapy for rectal cancer. *European Journal of Cancer*.

[27] Echteld M. A., van Zuylen L., Bannink M., Witkamp E., Van der Rijt C. C. (2007). Changes in and correlates of individual quality of life in advanced cancer patients admitted to an academic unit for palliative care. *Palliative Medicine*.

[28] Montassier E., Batard E., Massart S. (2014). 16S rRNA gene pyrosequencing reveals shift in patient faecal microbiota during high-dose chemotherapy as conditioning regimen for bone marrow transplantation. *Microbial Ecology*.

[29] Deng X., Li Z., Li G., Li B., Jin X., Lyu G. (2018). Comparison of microbiota in patients treated by surgery or chemotherapy by 16S rRNA sequencing reveals potential biomarkers for colorectal cancer therapy. *Frontiers in Microbiology*.

[30] Giloteaux L., Goodrich J. K., Walters W. A., Levine S. M., Ley R. E., Hanson M. R. (2016). Reduced diversity and altered composition of the gut microbiome in individuals with myalgic encephalomyelitis/chronic fatigue syndrome. *Microbiome*.

[31] Singh R., Jain S., Chabbra R., Naithani R., Upadhyay A., Walia M. (2014). Characterization and anti-microbial susceptibility of bacterial isolates: experience from a tertiary care cancer center in Delhi. *Indian Journal of Cancer*.

[32] Fentie A., Wondimeneh Y., Balcha A., Amsalu A., Adankie B. (2018). Bacterial profile, antibiotic resistance pattern and associated factors among cancer patients at University of Gondar Hospital, Northwest Ethiopia. *Infection and Drug Resistance*.

[33] Stringer A. M., Al-Dasooqi N., Bowen J. M. (2013). Biomarkers of chemotherapy-induced diarrhoea: a clinical study of intestinal microbiome alterations, inflammation and circulating matrix metalloproteinases. *Supportive Care in Cancer*.

[34] Tohamy S., Aboshanab K., El-Mahallawy H., El-Ansary M. R., Afifi S. (2018). Prevalence of multidrug-resistant Gram-negative pathogens isolated from febrile neutropenic cancer patients with bloodstream infections in Egypt and new synergistic antibiotic combinations. *Infection and Drug Resistance*.

[35] Villéger R., Lopès A., Veziant J. (2018). Microbial markers in colorectal cancer detection and/or prognosis. *World Journal of Gastroenterology*.

[36] Saltzman E. T., Palacios T., Thomsen M., Vitetta L. (2018). Intestinal microbiome shifts, dysbiosis, inflammation, and non-alcoholic fatty liver disease. *Frontiers in Microbiology*.

[37] Walker A. W., Sanderson J. D., Churcher C. (2011). High-throughput clone library analysis of the mucosa-associated microbiota reveals dysbiosis and differences between inflamed and non-inflamed regions of the intestine in inflammatory bowel disease. *BMC Microbiology*.

[38] Zhao Y., Lukiw W. J. (2018). Bacteroidetes neurotoxins and inflammatory neurodegeneration. *Molecular Neurobiology*.

[39] Zwielehner J., Lassl C., Hippe B. (2011). Changes in human fecal microbiota due to chemotherapy analyzed by TaqMan-PCR, 454 sequencing and PCR-DGGE fingerprinting. *PLoS One*.

[40] Gophna U., Konikoff T., Nielsen H. B. (2017). Oscillospiraand related bacteria - from metagenomic species to metabolic features. *Environmental Microbiology*.

[41] Naito Y., Uchiyama K., Takagi T. (2018). A next-generation beneficial microbe: Akkermansia muciniphila. *Journal of Clinical Biochemistry and Nutrition*.

[42] Reunanen J., Kainulainen V., Huuskonen L. (2015). Akkermansia muciniphila adheres to enterocytes and strengthens the integrity of the epithelial cell layer. *Applied and Environmental Microbiology*.

[43] Ye J., Lv L., Wu W. (2018). Butyrate protects mice against methionine-choline-deficient diet-induced non-alcoholic steatohepatitis by improving gut barrier function, attenuating inflammation and reducing endotoxin levels. *Frontiers in Microbiology*.

[44] Fijan S. (2014). Microorganisms with claimed probiotic properties: an overview of recent literature. *International Journal of Environmental Research and Public Health*.

[45] Lam Y. Y., Ha C. W. Y., Campbell C. R. (2012). Increased gut permeability and microbiota change associate with mesenteric fat inflammation and metabolic dysfunction in diet-induced obese mice. *PLoS One*.

[46] Forsyth C. B., Farhadi A., Jakate S. M., Tang Y., Shaikh M., Keshavarzian A. (2009). Lactobacillus GG treatment ameliorates alcohol-induced intestinal oxidative stress, gut leakiness, and liver injury in a rat model of alcoholic steatohepatitis. *Alcohol*.

[47] Thevaranjan N., Puchta A., Schulz C. (2017). Age-associated microbial dysbiosis promotes intestinal permeability, systemic inflammation, and macrophage dysfunction. *Cell Host and Microbe*.

[48] Xiao C., Beitler J. J., Higgins K. A. (2018). Associations among human papillomavirus, inflammation, and fatigue in patients with head and neck cancer. *Cancer*.

[49] Thomas A. M., Jesus E. C., Lopes A. (2016). Tissue-associated bacterial alterations in rectal carcinoma patients revealed by 16S rRNA community profiling. *Frontiers in Cellular and Infection Microbiology*.

[50] Li Y., Meng Q., Zhou B., Zhou Z. (2017). Effect of ensiled mulberry leaves and sun-dried mulberry fruit pomace on the fecal bacterial community composition in finishing steers. *BMC Microbiology*.

[51] Reddel S., Putignani L., Del Chierico F. (2019). The impact of low-FODMAPs, gluten-free, and ketogenic diets on gut microbiota modulation in pathological conditions. *Nutrients*.

[52] Hoehn R. S., Seitz A. P., Jernigan P. L., Gulbins E., Edwards M. J. (2016). Ischemia/reperfusion injury alters sphingolipid metabolism in the gut. *Cellular Physiology and Biochemistry*.

