# Supplementary material for: The Role of Gut Microbiome Perturbation in Fatigue Induced by Repeated Stress from Chemoradiotherapy: A Proof of Concept Study
Source: Adv Med. 2020 Feb 7;2020:6375876. doi: 10.1155/2020/6375876 (PMC7029262; doi:10.1155/2020/6375876)
Supplement: Supplementary Materials — S1 Table: comparison of alpha diversity indexes at the middle and end of CRT for participants based on chemotherapy treatment; end of treatment (5-Fu n=13; oral capecitabine n=9). S2 Table: comparison of alpha diversity indexes at the end of CRT for participants in each fatigue group(nonfatigue and fatigued N=7 and N=15). [file 6375876.f1.docx]

S1 Table. Comparison of alpha diversity indexes at the middle and end of CRT for participants based on chemotherapy treatment

| **Diversity Metric** | **5-FU** | **Capecitabine** | **T-test** | |
| --- | --- | --- | --- | --- |
|  | *Mean (SD)* | *Mean (SD)* | *T* | *p-value* |
| Shannon Diversity |  |  |  |  |
| Middle | 5.83+0.63 | 5.56+1.0 | 0.76 | 0.46 |
| End | 5.73+0.95 | 5.30+0.81 | 1.16 | 0.26 |
| Observed OTUS |  |  |  |  |
| Middle | 177.5+54.71 | 153.40+46.67 | 1.20 | 0.24 |
| End | 156.43+65.83 | 122.89+50.80 | 1.37 | 0.18 |
| Pielou |  |  |  |  |
| Middle | 0.79+0.06 | 0.77+0.09 | 0.59 | 0.56 |
| End | 0.81+0.09 | 0.78+0.05 | 1.09 | 0.29 |
| Faith Pylogenetic |  |  |  |  |
| Middle | 20.96+8.71 | 20.10+10.98 | 0.21 | 0.84 |
| End | 16.30+5.72 | 31.35+48.82 | -0.9278 | 0.38 |
| Middle of treatment (5-FU n=16; oral Capecitabine n=10); End of treatment (5-Fu n=13; oral Capecitabine n=9) | | | | |

S2 Table. Comparison of alpha diversity indexes at the end of CRT for participants in each fatigue group

| **Index** | **No-Fatigue** | **Fatigued** | **T-test** | |
| --- | --- | --- | --- | --- |
|  | **N=7** | **N=15** |  | |
|  | *Mean (SD)* | *Mean (SD)* | *T* | *p-value* |
| Shannon Diversity | 5.26+1.09 | 5.65+0.83 | -0.84 | 0.42 |
| Observed OTUS | 111.57+64.50 | 156.6+58.54 | -1.57 | 0.15 |
| Pielou | 0.81+0.08 | 0.78+0.08 | 0.94 | 0.36 |
| Faith Pylogenetic | 33.80+56.09 | 17.12+5.98 | 0.78 | 0.46 |
|  | | | | |
